# A Comprehensive Review of Antibiotics and Antimicrobial Resistance in the Aquaculture Sector of the World and Bangladesh

**DOI:** 10.1155/ijm/8818516

**Published:** 2025-12-04

**Authors:** Shama Afroze, Md. Faisal, Mohammed Nurul Absar Khan, Hrishika Barua

**Affiliations:** ^1^One Health Institute, Chattogram Veterinary and Animal Sciences University, Chattogram, Bangladesh; ^2^Department of Fishing and Post-Harvest Technology, Chattogram Veterinary and Animal Sciences University, Chattogram, Bangladesh

**Keywords:** AMR, antibiotic, aquaculture, public health, zoonotic bacteria

## Abstract

Antibiotics are molecules produced by a microbe to inhibit the growth of another microbe. Due to prolonged improper use, the situation in which these antibiotics do not work effectively on these microbes is termed antibiotic resistance or antimicrobial resistance (AMR). Aquaculture farming is one of the major industries in the world today due to the increasing consumption of seafood. Major antibiotics used in aquaculture farms include oxytetracycline, amoxicillin, ciprofloxacin, and azithromycin. The review paper has focused on the types and modes of action of major antibiotics, the mechanism of AMR, the dissemination of AMR in the ecosystem, and their impacts on human health. Moreover, it summarized the use of various antibiotics in aquafarms in Bangladesh and in different countries of the world. Due to the extensive use of these antibiotics, AMR has become a concerning public health issue all over the world. The article also tried to provide insights into the mechanisms of AMR of various pathogenic bacteria, which will help to develop new or modified antibiotics to fight against AMR. The knowledge regarding the rate of resistance and sensitivity of different antibiotics is essential and will provide baseline information for the treatment of these pathogenic bacteria.

## 1. Introduction

Antibiotics have long been believed to be a “miracle cure” for infectious diseases. Antibiotics are traditionally regarded as low-molecular-weight substances made by microbes that, at low doses, suppress microbial development [1]. Antibacterial, antifungal, antiparasitic, and antiviral medications are all included under the term “antimicrobial” [2]. Any natural, synthetic, or semisynthetic substance that can eradicate or prevent the growth of microbes (bacteria, fungi, or protozoa) falls under this definition [3]. For more than 70 years, bacterial infections have been treated using antimicrobials [4, 5]. Since antibiotics are antimicrobial substances that prevent bacterial growth or kill germs, they are crucial medications used in both human and animal healthcare to treat infections [6]. Recent research suggested that the overuse of antibiotics in aquaculture and other sectors results in the development of antimicrobial resistance (AMR) [7, 8]. According to a report of the World Health Organization (WHO) [9], AMR was directly responsible for 1.27 million deaths and contributed to 4.95 million deaths globally in 2019. AMR not only causes mortality and disability but also incurs large financial consequences. According to World Bank projections, AMR may lead to $3.4 trillion in annual gross domestic product (GDP) losses by 2030 and an additional $1 trillion in healthcare expenses by 2050 [10].

Aquaculture farming is a significant food industry sector that has grown at an average pace of 8.6%/year over the past few decades as a result of rising seafood consumption. Fish raised on farms made up 46% of all fish produced globally, with a worth of almost $250 billion [11, 12]. Since seafood animals can contract infectious diseases, aquaculture production has benefited from the use of antibiotics. Since aquaculture is expanding more quickly globally, it is essential to supply the rising demand for fish for human consumption. Because of rising demand and the degradation of natural supplies brought on by pollution and overfishing, aquaculture has replaced catch fisheries [13]. Increased production and nutrient pollution brought on by intensification create an environment that is conducive to the growth of diseases [14].

Bangladesh's aquaculture sector is thriving. With 2.73 million metric tons produced in 2021–2022, Bangladesh placed third in freshwater fish production, after China and India [15]. Currently, 62.5% of the fish produced worldwide for human consumption is produced through aquaculture [16]. Then, 4.70 million metric tons of fish are produced in Bangladesh each year, making up 2.43% and 22.14% of the nation's GDP from agriculture and other sources. For their daily survival, more than 12% of Bangladesh's population engages in full- and part-time fisheries and aquaculture operations [15]. The majority of cultural practices are enhanced-extensive and semi-intensive. Aquaculture practices also include the monoculture of pangas (*Pangasius pangasius*), tilapia (*Oreochromis niloticus*), shorputi (*Systomus sarana*), and Thai koi (*Anabas testudineus*). The ponds produce 4851 kg/ha of fish on average each year. Freshwater prawns (*Macrobrachium rosenbergii*) are also cultivated alongside carps in some regions of the nation. The two types of shellfish that are raised in the farms of Khulna, Bagerhat, Satkhira, and Cox's Bazar District are freshwater prawn (*M. rosenbergii*) and tiger shrimp (*Penaeus monodon*). About 2.54 lac MT of shrimp and prawns were produced overall in 2017–2018 [17, 18]. In some locations, this farming approach has also increased the frequent use of different medications and chemicals in aquaculture for health control [19]. Increased vulnerability to parasites, bacteria, fungi, viruses, and other illnesses can result from intensive fish farming, which is done to increase output. However, the improper and uncontrolled use of aqua medicines to treat illnesses creates possible threats to microbial resistance, which will eventually have an impact on both the environment and the industrial system [20].

Faruk et al. [21] stated that several antibiotics, such as oxytetracycline, amoxicillin, ciprofloxacin, sulfadiazine, chlortetracycline, and azithromycin, are most frequently used in pangas, tilapia, and other aquafarms of Bangladesh. Even while they have improved the health of countless numbers of people and animals, many antibiotics have also been losing their effectiveness since the dawn of the antibiotic era. Even as we create new antibiotics, bacteria have evolved defenses against them and are always creating new resistance. The rise of antibiotic resistance has received a lot of attention recently [22]. Researchers have studied a number of pathogens that affect fish and shellfish, including *Escherichia coli*, *Salmonella* sp., *Shigella* sp., *Aeromonas salmonicida*, *Aeromonas hydrophila*, *Aeromonas caviae*, *Aeromonas sobria*, *Edwardsiella ictaluri*, *Edwardsiella tarda*, *Photobacterium damselae piscicida*, *Vibrio cholerae*, *Vibrio vulnificus*, and *Vibrio parahaemolyticus*. Among these, a few are capable of zoonotic diseases [23, 24]. The rising issue of AMR is largely caused by the selection and spread of resistant organisms in developing countries, which are often associated with complex behavioral and socioeconomic antecedents [25]. Antibiotic resistance can spread globally as a result of globalization, endangering all countries [26]. Several bacteria, including those hazardous to human health, have been detected to exhibit resistance to all classes of antibiotics [27, 28]. Multidrug-resistant bacteria, which are bacteria that have become resistant to more than one type of antibiotic, as well as pan-drug-resistant bacteria, are becoming more and more common [29]. Infections that are resistant to antibiotics are challenging to treat and frequently fatal, which raises the rates of morbidity and mortality [30].

AMR is evolving into a very difficult issue in global public health. The focus of international efforts should be on finding a permanent solution to the AMR issue [27, 31, 32]. Antimicrobial-resistant organisms are thought to be the cause of more than 2 million infections and an almost 23,000 mortality rate each year, according to the US Centers for Disease Control and Prevention [31]. Multidrug-resistant bacterial infections are believed to be the cause of 33,000 annual fatalities in Europe [33]. According to a review on the worldwide crisis of AMR, commissioned by the UK government in 2014, if the rate of drug-resistant infections reached 100% by 2050, there would be an estimated 10 million deaths caused by drug-resistant infections [34, 35]. Without immediate action, by 2050, AMR could cause the world economy to lose more than $6 trillion in GDP, or approximately 4% of GDP [36]. Studies show that the prevalence of AMR in Bangladesh is due to widespread antibiotic abuse, non-human antimicrobial use, low drug quality, poor surveillance, and factors associated with personal and social determinants of poverty, including poor healthcare, malnutrition, chronic and recurrent infections, and the high cost of healthcare [36, 37].

Numerous authorities and organizations around the world have made this issue of global AMR a priority, and considerable investments have been made to address the problem of antibiotic resistance. The main goal of current mitigation methods is to reduce the use of antibiotics in human medicine, food, aquaculture, and other animal products. However, the AMR issue is more serious and complex than previously believed, necessitating the use of an upgraded surveillance and management method [38, 39]. This review study is aimed at analyzing the current knowledge on antibiotics and AMR, focusing on aquaculture and public health. This study focuses on the antibiotic usage patterns in aquafarms in Bangladesh in comparison with global practices. Furthermore, this study acts as a pathway to understand the consequences of AMR for human health.

## 2. History and Scope of AMR

Antibiotic-resistant microbes were already present in nature before the introduction of antibiotics, but they were largely absent from human flora [40]. Antibiotic-resistant bacteria have, however, increased alarmingly in prevalence over the intervening years. Researchers started to detect microbes that were resistant to the new antibiotics almost immediately after they were discovered. Ehrlich discovered drug-resistant trypanosomes even as early as 1909 when he first started researching dyes and arsenicals [41]. When penicillin was first used, the incidence of resistant *Staphylococcus aureus* strains in hospitals was less than 1%; by 1946, it had increased to 14%; by 1947, it had reached 38%; and now, it has reached more than 90% [41]. Over 80% of *S. aureus* strains worldwide exhibit resistance to both ampicillin and penicillin [42]. Four years after penicillin's widespread production began, it was discovered in 1947 that some strains of *Staphylococcus* were resistant to it. In the late 1940s, resistance to streptomycin, chloramphenicol, and tetracycline was also detected relatively quickly after their introduction. Since its discovery in Britain in 1961, methicillin-resistant *S. aureus* (MRSA) has become “very frequent” in hospitals around the world [43].

After World War II, sulfonamides were widely used in Japan as a treatment for *Shigella* infections. By 1952, only about 20% of *Shigella* isolates were sensitive to sulfonamide. As the Japanese began to use antibiotics such as chloramphenicols, tetracyclines, and streptomycins, multiply-resistant strains of *Shigella* rapidly emerged [44]. Sulfonamide lost its effectiveness as a treatment for meningococcal diseases within 30 years after its discovery [42]. Since then, more reports of resistance have been discovered, and almost all antibiotic-resistant microorganisms have been identified. As antibiotic resistance has reached a crisis point, some medical experts predict that we may soon be back to the deadly diseases of the preantibiotic era [40, 45, 46].

Multidrug resistance (MDR) has risen all throughout the world, posing a public health threat. Several recent studies have reported the emergence of MDR bacterial pathogens from various sources, including fish, birds, and cattle, emphasizing the need for routine antimicrobial susceptibility testing to detect the antibiotic of choice, as well as screening for emerging MDR strains. For instance, the high prevalence of MDR strains of *E. coli* was highly prevalent in the foot-and-mouth disease (FMD) outbreak in cattle in Damietta Province, Egypt. The study revealed that virulence (tsh, phoA, hly, eaeA, sta, and lt) and antibiotic-resistance genes (blaTEM, blaCTX, and blaKPC) of *E. coli* pose significant public health implications [47]. Again, several MDR bacteria were also identified from different samples. For example, MDR-*Mycobacterium avium* subsp. *avium* from domestic birds of Egypt, MDR-*E. coli* from aquatic environments of Bangladesh, MDR-*Proteus mirabilis* from healthy and diseased ducks of Egypt, MRSA causing bovine mastitis was detected in cattle in Egypt, MDR-*Pseudomonas aeruginosa* from fish samples (*O. niloticus* and *Clarias gariepinus*) of Egypt, and *Aeromonas veronii* isolated from freshwater fish (grass carp, carp, crucian carp, and koi carp) in Hebei Province were detected [48–53].

## 3. Classification of Antibiotics

Antibiotics are substances that either eliminate or stop bacterial growth. They may be created artificially or naturally by bacteria. More antibiotics have been found or created after Alexander Fleming's 1928 discovery of penicillin. More than 5000 antibiotics have been identified so far, and roughly 100 of those (belonging to 15 groups) are commonly utilized in treatment [54]. The commonly used commercial antibiotics and their classes with the target site have been summarized and represented in Table 1.

## 4. Major Antibiotics and Their Mode of Action

The antibiotic *chloramphenicol* is an antibiotic of broad spectrum that is produced through chemical synthesis, yet is naturally present in the body. It is a 70S ribosome inhibitor, thus preventing the formation of peptide bonds on the ribosome. This makes chloramphenicol an ideal medication for the treatment of eukaryotic and intracellular infections, such as those caused by chlamydia, as well as for the treatment of meningitis and intracerebrospinal fluid infections. However, it is not widely used due to potential fatal side effects, including aplastic anemia [41]. Chloramphenicol acetyltransferase is an enzyme that confers resistance to chloramphenicol. There are several of these enzymes that have been identified, and they all modify the chloramphenicol molecule to stop it from attaching to the bacterial ribosome. Chloramphenicol resistance is caused by alterations in the outer membrane of Gram-negative cells that impede the absorption of the medicinal product into the cell [56].

*Tetracycline* is another class of broad-spectrum antibiotics that inhibit the synthesis of bacterial protein. Tetracycline enters the cell through active transport and binds to the subunit 30S to inhibit the binding of aminoacyl-tRNA [57]. Resistance to tetracycline is caused by three pathways. Firstly, a multigenome efflux pump is synthesized to remove the medication as soon as it enters the cell. Secondly, several ribosome-protective proteins are functional to prevent the binding of tetracycline to ribosomes and the formation of resistance. Finally, a protein specific to the *Bacteroides* species is enzymatically inactivated by tetracycline inhibitors, which may allow the use of tetracycline, as well as combinations of such inhibitors, against bacteria that have previously become resistant to tetracycline [58].

*Sulfonamides* and *diaminopyrimidines* should be studied jointly because they both merely indirectly prevent the production of nucleic acids by preventing the synthesis of folate. A coenzyme called folic acid is required for the production of pyrimidines and purines. Although each of these pharmacological classes can be used on its own, they work better together. The enzyme that catalyzes the last stage in the manufacture of folate, dihydrofolate reductase, is inhibited by diaminopyrimidines, among which trimethoprim is the most widely used. [41].

A class of antibiotics known as *macrolides* is frequently employed to treat gram-positive and intracellular bacterial infections. The first of them was *erythromycin*, and since then, significant macrolides like *azithromycin* and *clarithromycin* have been found. Due to azithromycin's prolonged plasma half-life, some infections can be treated with a single dose, while others require a once-daily dose. *Clarithromycin* has improved absorption and causes less tummy pain [59]. The two main mechanisms of macrolide resistance are as follows: First, a drug removal device called an efflux pump has been discovered. Second, the ribosome can be altered to impart resistance. Allosterically preventing macrolide binding can result from mutations at several ribosomal locations, and dimethylation of one nucleotide on the 23S rRNA is a frequent change. This dimethylation not only inhibits the binding of macrolides but also confers resistance to the antibiotics streptogramin and lincosamide [59].

Another class of antibiotics called *streptogramins* prevents the formation of bacterial proteins, primarily in gram-positive bacteria. These antibiotics are essentially a blend of medications with distinct structural differences that work in concert. These substances cling to various locations on the 50S subunit. Streptogramins include the antibiotics named *pristinamycin*, *virginiamycin*, and *dalfopristin/quinupristin* [60].

*Quinolones* are a diverse group of broad-spectrum antibiotics that are frequently used to treat a wide range of illnesses, such as gonorrhea and anthrax. *Ciprofloxacin*, *norfloxacin*, and *nalidixic acid* are drugs in this class [61]. *Quinolone* resistance primarily arises from one of three methods. Reduced membrane porin expression is associated with resistance to various quinolones. These modifications also lead to cross-resistance to other medications that depend on these porins for activity. According to B. Normark and S. Normark [62], the production of efflux pumps in both gram-positive and gram-negative organisms represents a second resistance mechanism, and changing the target enzymes is a third. Both quinolone target proteins have been described as having a number of alterations that lower their binding affinities [63]. It is generally accepted that the development of high-level resistance to quinolones is the result of a sequence of sequential mutations in the relevant target genes, as opposed to a single mutation [62].

## 5. Types of AMR

The primary resistance mechanisms and the processes by which bacterial resistance develops must be understood. In general, AMR happens in the following ways.

### 5.1. Intrinsic AMR

Certain microbes possess intrinsic resistance to specific antimicrobials as a result of their structural or functional traits. For example, *P. aeruginosa* exhibits natural resistance to numerous antibiotics owing to its impermeable outer membrane [64].

### 5.2. Acquired AMR

Microorganisms gain resistance through genetic mutations or by obtaining resistance genes, such as those found in plasmids or transposons. However, the frequency of mutations that result in resistance is typically low, varies between species, and is influenced by a wide range of circumstances. For instance, MRSA obtains the mecA gene, which encodes modified penicillin-binding proteins [65].

### 5.3. Cross Antibacterial Resistance (Cross AR)

Cross AR denotes the occurrence in which bacteria acquire resistance to various antibiotics, frequently through overlapping mechanisms. This happens when resistance to a specific antibiotic grants resistance to another antibiotic, whether from the same class or a different one, owing to a shared resistance mechanism. Hooper [66] stated that if a bacterium becomes resistant to ciprofloxacin (a fluoroquinolone), it may also exhibit resistance to levofloxacin (another fluoroquinolone) as a result of mutations in the target enzyme (DNA gyrase).

### 5.4. MDR

MDR refers to resistance to three or more classes of antimicrobials. For instance, extensively drug-resistant *Mycobacterium tuberculosis* (XDR-TB) exhibits resistance to isoniazid, rifampin, fluoroquinolones, and injectable medications [67].

### 5.5. Extensively Drug-Resistant

Resistance of microbes to almost all existing antimicrobials is termed extensively drug resistant. For example, extensively drug-resistant *Acinetobacter baumannii* exhibits resistance to carbapenems, aminoglycosides, and polymyxins [68].

## 6. Mechanisms of Antibiotic Resistance

### 6.1. Enzymatic Degradation or Modification of Antibiotics

It is the most well-known resistance mechanism. This resistance is exclusive to a single drug class or specific to some of its members. Certain bacteria generate enzymes that neutralize antibiotics through degradation or chemical alteration. For example, *β*-lactamases act by hydrolyzing the *β*-lactam ring found in penicillin, cephalosporins, and carbapenems, which leads to a loss of their effectiveness [69].

### 6.2. Extrusion by Efflux Pumps

Bacteria use membrane proteins to actively pump antibiotics out of the cell. Certain resistant bacteria use an efficient transport system to push the antibiotic molecules out of the cell, in addition to absorbing the antibiotics as sensitive bacteria do, such as the *E. coli* AcrAB-TolC system, which exports tetracyclines and chloramphenicol [70].

### 6.3. Target Site Modification

Another process includes altering the target's structure; some bacteria modify the target site of antibiotics, which diminishes the binding efficacy of the drugs. For instance, mutations in DNA gyrase/topoisomerase lead to decreased binding of fluoroquinolones in *E. coli* and *Salmonella* [71].

### 6.4. Reduced Permeability

Bacteria alter their outer membrane structure to limit the penetration of antibiotics. For example, in *Klebsiella pneumoniae*, alterations in porins OmpK35 and OmpK36 reduce the uptake of carbapenem antibiotics, making it harder for the drug to enter and act against the bacteria [72].

### 6.5. Bypass Pathways

Bacteria can evolve alternative metabolic pathways that are not influenced by antibiotics. For example, enterococci, such as vancomycin-resistant enterococci (VRE), alter peptidoglycan precursors to diminish the binding efficacy of vancomycin [73].

### 6.6. Biofilm Formation

Some bacteria have the capabilities to generate biofilms. This provides a protective environment for bacteria against antibiotics through several mechanisms, such as the formation of a physical barrier created by extracellular polymeric substances, the presence of metabolically dormant cells known as persister cells, or increased horizontal gene transfer of resistance genes [74].

## 7. Administration Routes of Antimicrobial Agents

Antibiotics are administered via injection, which is the most efficient and direct method. On the other hand, the most popular method of administering antibiotics orally to fish is by combining them with food. Besides this, bath treatment is a well-liked approach to giving antibiotics. To get the required amount of response, nevertheless, it takes a lot more medication than injection and oral delivery [14, 75].

## 8. AMR Disseminated in Ecosystem

**Figure 1:**
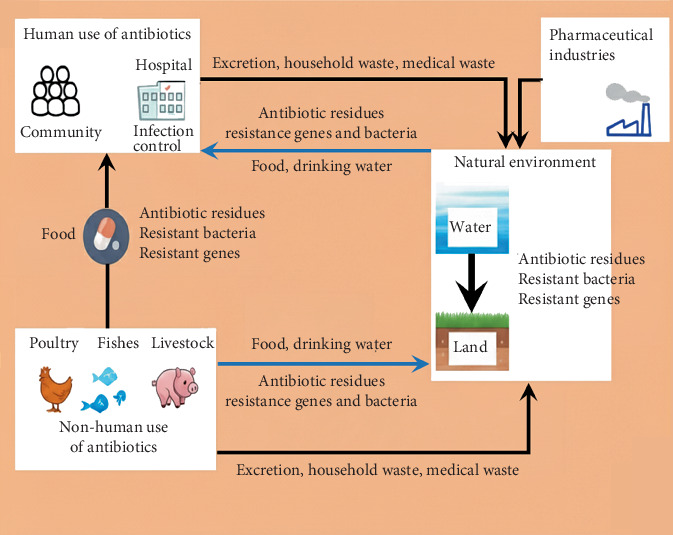
Dissemination of antibiotics within the ecosystem.

It has been acknowledged for a number of decades that the clinical setting is a key environment for AMR development. For a very long time, the principal AMR mitigation technique has been thought of as restricting the use of antibiotics during clinical therapy. Antibiotics, however, are also frequently employed in agriculture, particularly in the production of food for animals, for growth promotion, prevention, and therapy. According to reports, just 7.7 million pounds of antibiotics were purchased for human use over the same time in the United States, as opposed to 29.9 million pounds for the production of meat and poultry [76]. Antibiotic use in clinical settings is thus an essential but not the only factor in the emergence of AMR, and the problem of resistance is more nuanced than previously believed. Since 2005, findings from numerous studies have provided strong evidence pointing to the involvement of numerous risk factors in the emergence, enrichment, spread, and persistence of AMR [77, 78]. Dissemination of antibiotics within the ecosystem [79] is represented in Figure 1.

## 9. Antibiotic Usage in World Aquaculture

Sporadic outbreaks of infectious illnesses have slowed down the aquaculture industry's rapid expansion. It was inevitable that some aquaculture production would be lost due to disease outbreaks. Disease-related losses to China's aquaculture industry in 2010 totaled 295,000 MT [80]. Disease outbreaks were frequently caused by unsanitary and stressful conditions that existed in aquaculture environments with high stocking densities. Antibiotics have been utilized in aquaculture as therapeutic and/or prophylactic treatments to reduce mortality loss and prevent and treat bacterial infection [23]. In general, industrialized countries have imposed more stringent restrictions on the utilization of antibiotics in the aquaculture sector than developing countries, which are responsible for the majority of aquaculture production. In the United States, the FDA has approved four distinct types of antibiotics for aquaculture use (Table 2).

Most of the time, due to financial constraints, a sizable proportion of aquaculture facilities globally and some aquaculture farmers utilize antibiotics improperly. The use of antibiotics is not always tracked and is limited by regulatory organizations in some nations. The most often used antibiotics throughout the 20 nations that produce aquaculture were oxytetracycline, oxolinic acid, and sulfa/trimethoprim, which were used in 17 and 13 countries, respectively. With antibiotic usage reaching 22, 21, 14, and 14, respectively, Japan, Vietnam, Thailand, and China all used more antibiotics than those countries [55]. It is well known that using antibiotics improperly in aquaculture causes a number of challenges, such as bacterial resistance, antibiotic residues in food product tissues and the aquaculture environment, and the expense of researching and averting unforeseen consequences. Antibiotic residues have been found in the aquaculture environment and in aquaculture products on numerous occasions. Trimethoprim, sulfamethoxazole, norfloxacin, and oxolinic acid were found at amounts ranging from 1.04 to 820.49 ppm in water and muck in shrimp ponds and nearby canals [82]. Sulfonamides were found to be the most prevalent antibiotics in the water, according to 132 samples from Baiyangdian Lake in China, whereas quinolones were more prevalent in sediments, aquatic animals, and plants [83].

Specifically, from Vietnam, Bangladesh, China, Indonesia, India, Chile, and Taiwan, antibiotics like fluoroquinolones, nitrofurans, and chloramphenicol were the most frequently found antibiotic residues in imported aquaculture goods between 2004 and 2007 [84]. The formation of AMR bacteria and antimicrobial resistance genes (ARGs) in the environment, food products, and humans exposed to such environments and food would typically be caused by antibiotic residues in the food items as well as the environment, at lower concentrations but over longer periods. Additionally, eating food products that contain antibiotic residues may change the natural gut microflora [55].

## 10. Antibiotic Use in Aquaculture of Bangladesh

Due to ignorance, aquaculture farmers are unable to continuously maintain the prescribed amount, and the indiscriminate use of medications may cause a significant loss in the biodiversity of aquatic creatures. Aquaculture in Bangladesh uses a variety of medications and chemicals, depending on farmers' desire and market availability [85, 86]. However, nothing is known about the list of medications that have been licensed for use in aquaculture. Aquaculture medicine approval may be hampered by a difficult licensing process. Farmers claimed that sulfadiazine and oxytetracycline were two antibiotics that were frequently used in aquaculture to treat conditions including vibriosis and ulcerative disorders [87].

According to research by Faruk et al. [21], farmers mostly employed antibiotics from six distinct groups based on active components to treat sick fish. These antibiotics were often administered orally by mixing them with feed or pond water (Table 3). Antibiotics were found to be the most popular, with 25.59% of the population using them. Amoxicillin was the second most popular, with 25.05%, followed by a few others with lower selling rates. Ciprofloxacin was the third most popular, with 17.79%, followed by sulfadiazine with 14.68%. A few others with lower sales rates were chlortetracycline with 6.37% and azithromycin with 5.26%. The pharmacies that sell the antibiotics are run by seven different companies, including Renata, ACI Animal Health, Novartis, Eon, Navana Pharma, Acme, and SK+F. There was a total of 15 antibiotics in the trade-named group, with five antibiotics, oxytetracycline being the main active ingredient [21].

A broad-spectrum antibiotic, the amoxicillin group, has been discovered to be effective against both gram-positive and gram-negative bacteria in fish. Farmers found that amoxicillin was a reasonably effective medication for treating fish streptococcal infections. The term “Acimox” refers to two antibiotics, Acimox and Renamox, both of which contain the active component amoxicillin trihydrate. Three antibiotics included in the ciprofloxacin class were Renaflox, ciprofloxacin plus, and ciprofloxacin-vet. The most often utilized ciprofloxacin by fish farmers was found to be Renaflox. However, it was noted that it is only authorized for veterinary uses, not aquaculture, at the product level. The active pharmaceutical ingredient of ciprofloxacin is ciprofloxacin hydrochloride, as defined by the United States Pharmacopeia (USP). The farmers also utilized a variety of water doses of three sulfadiazine medications (Ati-vet, Micronid, and Eskatrim-vet) [21, 88].

Sulfadiazine-based antibiotic preparations consisted of a blend of the active components, such as sulfadiazine, trimethoprim, and erythromycin. In the drug stores, chlortetracycline with the brand name “Captor” and the active components British Pharmacopoeia (BP) 45% hydrochloride and chlortetracycline were to be found. Farmers discovered that Azin-vet was the only antibiotic in the azithromycin class that they could use against bacterial illnesses at a much lower dose, such as 20 mg/kg body weight of fish [21, 88–90]. The use of antibiotics, growth promoters, probiotics, and other chemicals [91] for the pond preparation and disease treatment of catfish, carps, and shellfish farms in the different districts and regions of Bangladesh over the last decade has been summarized in Table 4.

## 11. Worldwide AMR in the Aquaculture System

The aquaculture system has reportedly been linked to a number of AMR pathogens (Table 5), including *Vibrio*, *Salmonella*, *Aeromonas*, and *E. coli* [103, 105, 106]. Additionally, significant concentrations of AMR bacteria linked to a variety of commensal bacteria have been discovered in a variety of aquaculture products and farm production systems [107–109]. According to study trends, AMR microorganisms, such as MDR bacteria, were widespread in aquaculture.

## 12. AMR in the Aquaculture Practices in Bangladesh

In Bangladesh, it was not unusual to find bacteria resistant to antibiotics in freshwater fish and aquaculture systems, but to date, very few studies have highlighted the findings of AMR in the aquaculture sector. Hossain et al. [110] reported the isolation of different bacteria, such as *Vibrio* sp., *E. coli*, *Pseudomonas* sp., and *S. aureus*, from different fish samples, of which 80% were resistant to fluoroquinolones and sulfanilamide and 100% resistant to five major antibiotics (macrolides, penicillins, tetracyclines, aminoglycosides, and cephalosporins). In another study, ampicillin was found to be resistant to *V. parahaemolyticus* and *Listeria* [111, 112]. Thornber et al. [113] stated that amoxicillin resistance was up to 34% against *Aeromonas* bacteria. *S. aureus* and *E. coli* showed resistance against erythromycin [111]. Furthermore, resistance to erythromycin, cephradine, sulfamethoxazole, and chloramphenicol was demonstrated by *Pseudomonas fluorescens* [114].

## 13. AMR in Some Zoonotic Bacteria

It is impossible to overstate the impact that zoonotic illnesses spread by fish and water have had on populations around the world. Malnutrition and mortality are still brought on by digestive illnesses in underdeveloped nations. *Aeromonas* spp., *Campylobacter* spp., *Salmonella* spp., *Shigella* spp., *Vibrio* spp., and *E. coli* are a few of the most significant zoonotic diseases associated with aquaculture. Antibiotic resistance is becoming a bigger issue in the treatment of the diseases these species cause, and they spread sickness all over the world [115–119].

### 13.1. *Salmonella* spp.

*Salmonella* spp. are gram-negative rod-shaped microorganisms that live in the intestinal tract of various mammals, reptiles, and birds. They can live for an extended amount of time in food, earth, and water [120]. Typhoid fever and salmonellosis are the two main illnesses connected to *Salmonella* spp. Although the prevalence of typhoid fever has considerably dropped in wealthy nations, there are still 600,000 deaths and 16.6 million cases worldwide each year, with the majority occurring in underdeveloped nations [121]. *Salmonella* infections have generally been treated with ampicillin and chloramphenicol [121]. However, since 1988, ciprofloxacin has been the most often recommended medication for these infections [120]. By the end of the 1960s, multiple medication resistance was widespread in infections with nontyphoidal *Salmonella*, and it has significantly risen since then. The first known isolation of the multiresistant *Salmonella typhimurium* 18 strain, DT104, was made in England in 1988 [122]. Then, 90% of the human DT104 isolates discovered during the ensuing 10 years were discovered to be MDR, meaning they were resistant to chloramphenicol, ampicillin, streptomycin, tetracycline, and sulfonamides. Since then, this strain has spread to many other countries in the world, including Europe and the United States. Since 1997, DT104 isolates have developed fluoroquinolone and trimethoprim resistance. Multiplicity-resistant *Salmonella typhi* cases have been reported more frequently globally since 1986 [122, 123].

### 13.2. *Shigella* spp.

*Shigella* spp. are gram-negative, rod-shaped, and closely related to *Escherichia* species. *Shigella flexneri*, *Shigella sonnei*, *Shigella dysenteriae*, and *Shigella boydii* are the four pathogenic species. Most cases of shigellosis in the United States are brought on by *S. sonnei*, whereas *S. flexneri* is the most prevalent worldwide. Shigellosis affects 165 million people annually worldwide, almost all of whom live in underdeveloped nations. In contrast, in the United States, the number of cases is 20–30,000 per year. Shigellosis is the leading cause of death worldwide (1.1 million in 2010). Then, 69% of cases occur in children under 5 years of age. Poor sanitation and lack of clean drinking water are the primary causes of transmission in poor countries [124]. Antibiotic resistance was first identified in Japan, where diarrhea caused by shigellosis was a major health problem. By 1950, 80%–90% of shigellosis isolates were sulfonamide resistant. By 1965, shigellosis had developed resistance to several antibiotics [125]. These antibiotics include ampicillin, triflocin, chloramphenicol, and streptomycin. Resistance to shigellosis has been steadily increasing since. In Israel, there is currently 85% resistance to ampicillin, trimethoprim is 94% resistant, and tetracycline is 87% resistant. In 2000, there was a rise from 23% to 87%. There have also been reports of ciprofloxacin resistance for the *Shigella* species [126].

### 13.3. *Aeromonas* spp.

The motile aeromonad, particularly *A. salmonicida*, is a significant fish disease in aquaculture. *Aeromonas* is resistant to a range of antibiotics, with oxytetracycline resistance reported to be between 58% and 83% in a study of catfish farms isolated from aeromonads [127]. Similarly, a study of various Danish farms isolated aeromonads found that 69% of the samples were resistant to the active metabolite oxytetracycline. Additionally, a study conducted by Schmidt et al. [128] found aeromonads to be resistant to 43% of sulfadiazine and 20% of oxolinic acids. Reports from Scottish farms suggest that resistance to oxytetracycline is between 50% and 55%, while resistance to oxolinic acids is between 31% and 54%. On the other hand, sediments in Italy surrounding fish farms have been found to have a higher level of resistance to a variety of antibiotics, such as tetracycline, ampicillin, and streptomycin [129].

### 13.4. *Vibrio* spp.

The genus *Vibrio* is composed of gram-negative bacteria with a comma-like curved shape. Several species of *Vibrio* are capable of causing foodborne infections, typically due to consumption of undercooked seafood, and are known to have a high salt tolerance, allowing them to survive in freshwater [22]. In a study conducted in 1998 and 2000, 18% of *V. cholerae* isolated from Chesapeake Bay and its watershed were found to have antibiotic resistance to erythromycin, polymyxin, streptomycin, and tetracycline, according to McNicol et al. [130]. Moreover, 25.9% of the isolates were resistant to ampicillin and tetracycline, and 73% to streptomycin [131]. Two hundred fifty-six strains of *V. cholerae* obtained between 1998 and 2000 were tested for antibiotic resistance in order to identify natural antibiotic resistance patterns [22, 132].

### 13.5. *E. coli*

An extremely prevalent gram-negative rod typically found in mammal digestive tracts is *E. coli*. The prevalence of drug–drug resistance was estimated to be between 21% and 85% for sulfonamide, between 17% and 72% for ampicillin, between 24% and 60% for tetracyclines, and between 1% and 4% for trimethoprim [42]. For many years, fluoroquinolone resistance was uncommon, but since the early 1990s, it has become more common. More than a third of the isolates in one study conducted in Spain were found to be resistant to three or more additional antibiotics, bringing the frequency of resistance to 22% [133]. Fluoroquinolone resistance is found in 11.3% of Taiwanese isolates, and reduced susceptibility is observed in 21.7% of isolates. Unsurprisingly, it is thought that decreased susceptibility will increase the occurrence of resistance [134].

## 14. Impact of AMR on Public Health

**Figure 2:**
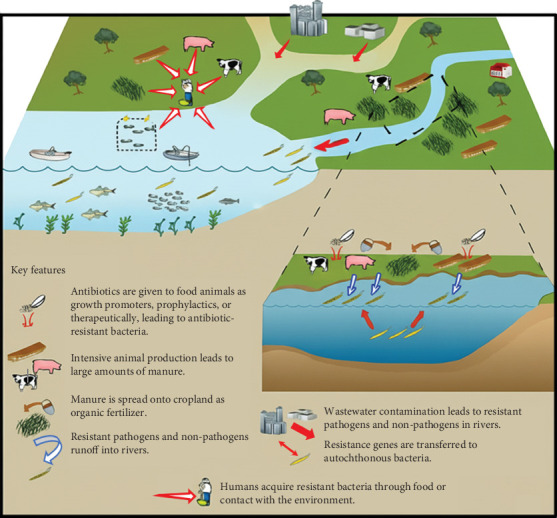
Animal–environment–human web for the transmission of AMR bacteria.

Antibiotics in aquaculture have been linked to the development of diseases in fish and shellfish that are resistant to different types of antibiotics, which can lead to epidemics that are hard to treat. The development and spread of drug-resistant pathogens debilitate our capacity to treat common diseases and to perform life-saving strategies, including cancer chemotherapy and cesarean section, hip replacements, organ transplantation, and other surgeries [9, 135–137]. AMR could also be spread to the general public through the ingestion of seafood using aquaculture products, depending on how well food processing works to inactivate microbes. Both clinical isolates and aquaculture isolates have been discovered to contain specific AMR determinants. For instance, substantial similarity (92%–100%) was found between the microbial resistance genes found in isolates from Japanese fish farms and the corresponding genes reported in clinical isolates [138]. Drug-resistant diseases also have an adverse effect on plant and animal health, lower agricultural output, and jeopardize food security. The causes and effects of AMR particularly affect vulnerable groups and those who live in low-resource environments [9]. AMR *Aeromonas* sp. and *E. coli* transmission occurs between human and aquaculture habitats, as well as in places as diverse as Norway, Scotland, England, and Germany [139]. Fish and other aquatic animals are unusual in that they are continually exposed to microorganisms from their diet and the environment in which they are produced (water, soil, etc.), which affects their gut microbiota and has additional effects on the microbiome of the water system in which they reside.  Figure 2, which was modified from Gangle [22], depicts the intricate network between humans, animals, and the environment that contributes to the spread of AMR bacteria.

*Salmonella*, *E. coli*, *V. cholerae*, *V. parahaemolyticus*, *V. vulnificus*, *Shigella*, *Aeromonas*, and *Streptococcus* are pathogens that can infect people and cause zoonotic infections that are resistant to antibiotics [115–119]. Looney et al. [140] stated that the occurrence of other fish-related water bacteria as possible human pathogens has been of concern, as it can cause opportunistic infections, particularly among the immunocompromised, and emphasizes the need to monitor environmental reservoirs and introduce effective infection-controlling measures. Antibiotic-resistant bacteria have been hypothesized to have been selected from the normal intestinal flora of fish due to the presence of antibiotics in the aquaculture industry. It is possible that the AMR markers present in the typical aquatic flora may be the source of resistance markers in aquatic infections, similar to those found in land-based animals and humans [141, 142], and may be becoming zoonotic (Table 6). As aquatic bacteria share a broad range of antibiotic susceptibility genes with terrestrial bacteria, the potential implications for human health may be particularly relevant in countries with intensive, preventive, and unregulated aquaculture practices [143, 144].

## 15. AMR on Human Health Originating From Fisheries in Bangladesh

The integrated culture of fish and poultry is widely practiced in Bangladesh, which poses a serious threat to public health. Due to these types of farming systems, AMR within aquaculture environments is increasing day by day. Metagenomic analysis of various environmental samples from the integrated culture system revealed that a total of 384 ARGs were present in that system, among which tetracycline resistance genes (*tetM* and *tetX*) were abundant. Fish and chicken are among the most important sources of protein and are extensively used in the Bangladeshi diet. The study reveals that contamination of MDR bacteria or traces of antibiotics in the food samples can cause fatality in humans after consumption [145]. Again, in another study on raw seafood, the presence of antibiotic-resistant and virulent *Enterococcus faecalis* was proven in the samples. Among the identified isolates, 88.6% demonstrated biofilm-forming abilities, including 25% strong and 63.6% intermediate biofilm formers, indicating an alarming situation given the enhanced persistence and resistance associated with biofilms. Consumption of such seafood that is infected with MDR, virulent, and biofilm-forming *E. faecalis* will result in difficult-to-treat infections like bacteremia, urinary tract infections, and endocarditis in humans [146].

In another study on seafood, the presence of extended-spectrum beta-lactamase (ESBL)–producing *E. coli* was reported, which can cause a severe threat, like urinary tract infections, septicemia, and other invasive diseases to humans through the food chain. In that study, all the 102 isolates of *E. coli* were resistant to ampicillin, and 69.8% of isolates exhibited MDR [147]. Again, MDR-*E. coli* with blaTEM, blaSHV, blaCTX, and biofilm-forming genes like fimC were found in 97% of cultured fish in Mymensingh, Bangladesh. The resistant bacteria could be incorporated into the human food chain, exposing an individual to a hard-to-treat infection [148]. Meanwhile, extensive aquaculture practices have led to the accumulation of antibiotic residues like ciprofloxacin, oxytetracycline, chlortetracycline, levofloxacin, and enrofloxacin in fish species that are highly consumed (such as tilapia, stinging catfish, climbing perch, and pabda) due to antibiotics. Although the immediate toxicological risk is not critical, these residues allow the selection of resistant bacteria in the human gut and foster the transmission of AMR [149].

## 16. Action Plans and Policies to Prevent Antibiotic Resistance

The transmission of AMR can take place across borders through the export of goods and international travel, as well as through environmental media such as soil or water [26]. In 2015, the WHO adopted a global action plan on AMR, aiming to promote modern medicine as a viable option for treating and preventing diseases [150]. To safeguard certain antibiotics essential for human health, in 2017, the WHO issued guidelines regarding the use of medically relevant antimicrobials in food-producing animals. These guidelines proposed a reduction or prohibition of certain antibiotics, such as streptothricins, glycopeptides, and colistin, in the agricultural sector. Streptothricins, including nourseothricin, were linked to human toxicity and rapid development of plasmid-mediated resistance in *E. coli* of treated swine that, in turn, spread to humans. Likewise, the application of avoparcin in poultry and pigs caused the selection of the VanA gene cluster, which gave rise to glycopeptide-resistant enterococci that were transmitted to humans, presumably via the food chain. Colistin, a common livestock drug, enabled the appearance of the new mcr-1 resistance gene in animals that have progressively begun to be detected in human isolates [25]. Many countries, particularly the United States and the EU, have developed their own national AMR prevention and control plans and strategies in accordance with the WHO's recommendations [30]. In response to the threat of AMR, in 2015, the Australian government developed its first national AMR strategy [151].

The emergence of AMR in developing countries, including Bangladesh, has led to the development of a number of laws, regulations, and ordinances in the country. Out of these, seven have been specifically addressed to AMR, including three for humans, four for animals, and the environment. Since the update of the National Drug Policy in 2016, the government of Bangladesh has developed three important policy documents and guidelines. These cover a range of topics related to the prevention and control of AMR in healthcare settings, such as an ADR policy for monitoring the sale and dispensing of drugs without a prescription of 2017, an STG for the appropriate use of antimicrobials in subdistricts, and a guideline for the use of antimicrobial agents in treatment. Moreover, Bangladesh's 2021–2026 National Strategy and Action Plan for Antimicrobial Resistance Containment (ARC) was created with technical assistance from the USAID Medicines, Technologies, and Pharmaceutical Services (MTaPS) Program. The proposal was authorized by the National Technical Committee for ARC of the Government of Bangladesh in March 2022, marking a significant advancement in the fight against AMR [152].

Livestock development policy was set up by the MoFL in 2007 to tackle AMR in the industry, but it found that there were not enough veterinary services and the regulatory framework was not being properly implemented. In 2010, new laws were put in place to control AMR in food animals and fish, banning things like antibiotics, hormones, and pesticides. A person who violates this law could get a fine of up to BDT 50,000 or up to a year in jail ($650). The documents addressed various issues, such as the need for an antibiotic prescription to be purchased, the recommended antibiotics for a specific system's ailments, and the initial and secondary lines of antibiotic therapy for specific pathogens. Livestock-related documents emphasize the prohibition of the use of antibiotics in general as a growth agent. Finally, the Ministry of Health and Family Welfare's (MoHFW) Disease Control Unit (DCU) has developed a National Plan for Reducing Amyloid Acute Respiratory Disease (AMR) in Bangladesh from 2017 to 2022, with a plan for implementation in line with the global plan [153]. The document emphasizes the development of standardized treatment protocols, antimicrobial stewardship, reference laboratories, good manufacturing practice (GMP), and good pharmacy practice (GPP) standards, as well as the implementation of comprehensive surveillance measures to guarantee the prudent utilization of antibiotics across all areas.

## 17. Conclusions

This study was aimed at providing insights into the AMR of major pathogenic bacteria, which has emerged as a serious and growing threat to human health worldwide. The inappropriate use of various antibiotics in the aquaculture and seafood sectors of different developed and developing countries is patronizing the AMR. This resistance is not only for the common microbes but also for zoonotic bacteria. The indication of AMR pathogens in Bangladesh, as well as all over the world, is now a challenging public health issue, and proper initiatives are required to solve this problem. Understanding the level of resistance and susceptibility of different antibiotics is crucial for the development of novel or modified antibiotics to save human beings. By focusing on the types of antibiotics and modes of action of major antibiotics and their impacts on human health, this study provided baseline information to fight against AMR.

## Figures and Tables

**Table 1:** List of antibiotics used in therapeutic treatment and their targets [55].

**Antibiotic class**	**Example**	**Target**
*β*-Lactam	Penicillin, cephalosporin, penem, and monobactam	Peptidoglycan synthesis
Glycopeptide	Vancomycin and teicoplanin	
Aminoglycoside	Gentamicin, streptomycin, and spectinomycin	Translation
Tetracycline	Oxytetracycline, minocycline, and tigecycline	
Macrolide	Erythromycin and azithromycin	
Lincosamide	Clindamycin	
Streptogramin	Synercid	
Oxazolidinone	Linezolid	
Phenicol	Chloramphenicol	
Quinolone	Ciprofloxacin	DNA replication
Pyrimidine	Trimethoprim	C1 metabolism
Sulfonamide	Sulfamethoxazole	
Rifamycin	Rifampin	Transcription
Lipopeptide	Daptomycin	Cell membrane
Cationic peptide	Colistin	

**Table 2:** Antibiotics approved for use in aquaculture [81].

**Approved antibiotics**	**Administration route**	**Species/class**
Oxytetracycline hydrochloride	Immersion	Finfish fry and fingerlings
Florfenicol	Oral via feed	Salmonids, catfish, and freshwater-cultured finfish
Oxytetracycline dihydrate	Oral via feed	Salmonids, catfish, *Oncorhynchus mykiss*, and lobster
Sulfadimethoxine/ormetoprim	Oral via feed	Catfish, salmon, and trout
Formalin	Immersion	Finfish and their eggs, penaeid shrimp, salmon, trout, catfish, and bluegill
Chorionic gonadotropin	Injection	Brood finfish
Tricaine methanesulfonate	Immersion	Ictaluridae (catfish), Salmonidae, Esocidae, and Percidae
Sulfamerazine	Oral via feed	Trout (rainbow, brook, and brown)

**Table 3:** Summary of antibiotics with trade name and active ingredients [21].

**Antibiotics (group)**	**Trade name**	**Active ingredients**	**Sources (company)**	**Dose**
Azithromycin	Azin-Vet	Azithromycin USP	Acme Laboratories Ltd.	20 mg/kg body wt. for 3–5 days
Chlortetracycline	Captor	Chlortetracycline hydrochloride BP 45%	Novartis Animal Health Ltd.	2.5–3.5 gm/kg feed for 3–5 days
Amoxicillin	Acimox	Amoxicillin trihydrate BP	Navana Pharma. Ltd.	40 mg/kg body wt. for 5–10 days
	Renamox	Amoxicillin trihydrate 300 mg/gm	Renata Pharmaceuticals Ltd.	1 gm/kg feed for 5–7 day
Ciprofloxacin	Renaflox	Ciprofloxacin HCL USP	Renata Pharmaceuticals Ltd.	1.5–2.5 gm/kg feed
	Cipro-plus	Ciprofloxacin 5.50 mg	Novartis Animal Health Ltd.	2–4 gm/kg feed
Oxytetracycline	Renamycin	Oxytetracycline hydrochloride USP	Renata Animal Health Ltd.	50 mg/kg body wt.
	Aquamycin	Oxytetracycline+hydrochloride	ACI Animal Health Ltd.	40 mg/kg body wt.
	Oxysentin	Oxytetracycline+HCL BP	Novartis Animal Health Ltd.	1–2 gm/kg feed for 5–7 days
	Oxy doxy-F+hydrochloride 20%	Oxytetracycline	ACI Animal Health Ltd.	0.25 gm/kg body wt. for 2 times/day
	Oxy-D-Vet	Oxytetracycline 20%+doxycycline 10%	Eon Animal Health Ltd.	5–10 gm/kg body wt.
Sulfadiazine	Eskatrim-Vet	Sulphadiazine+trimethoprim	SK+F	0.1 mL/L water for 2–3 days
	Micronid	Erythromycin and sulphadiazine+trimethoprim	Renata Pharmaceuticals Ltd.	0.5–1 gm/L water
	Ati-vet	Sulfadiazine+trimethoprim	Acme Laboratories Ltd.	0.1 mL/L water for 2–3 days

**Table 4:** List of aqua medicines, antibiotics, and chemicals used in the last decade in aqua farms.

**Years**	**Study area**	**Total aqua medicines**	**Antibiotics**	**Oxygen**	**Growth promoter**	**Disinfectants**	**Raw chemicals**	**Probiotics**
2020	Noakhali	49	2	7	16	2	—	9
2020	Patuakhali	71	10	9	10	9	—	2
2020	Rangpur	81	9	10	10	9	7	8
2020	Bogura	79	11	10	8	12	—	9
2020	Moulvibazar	46	5	12	11	5	—	—
2020	Southern coastal region	17	2	3	2	2	—	3
2020	North-west region	4	—	—	—	—	4	—
2019	North Chattogram	75	13	10	10	10	—	6
2019	South-western region	65	7	7	13	6	—	5
2019	Jashore	1	—	—	—	—	1	—
2019	Mymensingh	5	—	—	—	—	5	—
2019	Mymensingh	5	—	—	—	—	5	—
2019	Jhenaidah	38	8	6	7	5	5	—
2018	Jashore	47	5	6	8	7	—	—
2018	Jamalpur	53	9	7	20	6	—	—
2018	Mymensingh	3	—	—	—	—	2	—
2018	Jashore	35	3	—	—	6	3	—
2018	Jashore	2	—	—	—	—	2	—
2017	Mymensingh	1	1	—	—	—	—	—
2017	Lalmonirhat	6	—	—	—	—	6	—
2017	Comilla	143	19	20	27	18	—	11
2017	Shylhet	53	8	10	10	10	—	—
2017	North-west region	1	—	—	—	—	1	—
2017	Brahmanbaria	1	—	—	—	—	1	—
2017	Jashore	3	—	—	—	—	3	—
2016	Bagerhat	5	—	—	—	—	5	—
2016	Jashore	4	—	—	—	—	4	—
2016	Jashore	4	—	—	—	—	1	—
2015	Jashore	3	—	—	—	—	3	—
2015	Shylhet	69	8	8	14	7	—	—
2015	North-eastern region	52	5	6	5	5	6	—
2015	Narail	24	3	3	4	3	—	—
2014	Satkhira	35	5	4	6	4	—	5
2014	Patuakhali	38	7	7	—	7	—	—
2014	Mymensingh	45	8	8	5	10	—	—
2014	Khulna and Bagerhat	46	12	10	—	8	—	—
2014	Bogura	32	—	—	—	6	—	—
2012	South-west region	122	15	18	26	12	—	13
2011	Mymensingh	43	11	5	3	14	—	—

**Table 5:** AMR observed in seafood and aquaculture in different countries.

**Bacterial genera**	**Country**	**Antibiotic class**	**Source of bacteria**	**References**
*Vibrio* and *Aeromonas*	Australia	AMP, AMX, CXN, CEF, CF, CHL, FLO, NA, OA, GT, KN, ERY, TET, OXY, TMP, SXZ, and T-S	Farmed fish, crustaceans, and water	Akinbowale et al. [92]
*Pseudomonas* and *Aeromonas*		AMX, CEF, CF, TI, OXY, FLO, S, SXZ, CT, OA, CHL, TMP, and NIT	Farmed fish and sediment	Akinbowale et al. [93]
*Vibrio parahaemolyticus*	China	AMP, S, RIF, and SPT	Shrimp	He et al. [94]
*Salmonella*		AMP, AMC, CEF, CHL, TET, NAL, S, KN, and CZL	Fish, shrimp, and oysters	Yang et al. [95]
*Aeromonas hydrophila*		AMP, CIP, KN, NAL, S, and TET	Fish	Yang et al. [96]
*Salmonella*		AMP, ERY, GT, NAL, NIT, PEN, S, TET, and TMP	Fish	Broghton and Walker [97]
*Pseudomonas* and *Serratia*	Denmark	AMP, ERY, AMC, CEZ, T-S, TET, CF, and CT	Fish and shrimp	Uddin et al. [98]
*Vibrio parabrhaemolyticus*	Korea	AMP, CEF, S, ERY, RIF, and VAN	Oysters	Kang et al. [99]
*Bacillus*, *Staphylococcus*, and *Acinetobacter*	Malaysia	S, AMP, PEN, ERY, CEF, RIF, NIT, PIP, CRO, and CEZ	Fish, shrimp sediment, and water	Kathleen et al. [100]
*Vibrio parahaemolyticus*	India	AMP, AMX, CAR, CPD, CEF, COL, and S	Fish	Sudha et al. [101]
*Escherichia coli*	South Korea	TET, S, CIP, AMP, T-S, TI, NAL, KN, and CHL	Fish, mollusks, and crustaceans	Ryu et al. [102]
*Escherichia* and *Staphylococcus*	Switzerland	CIP, NAL, TET, SXZ, AMP, TMP, CHL, PEN, KN, and GEN	Fish, shrimp, and oysters	Boss et al. [103]
*Pseudomonas* and *Aeromonas*	Vietnam	AMP, T-S, CHL, NIT, NAL, CIP, NOR, TET, DOX, GT, S, and KN	Fish, water, and sediment	Nguyen et al. [104]

Abbreviations: AMC, amoxicillin–clavulanic acid; AMP, ampicillin; AMX, amoxicillin; CAR, carbenicillin; CEF, cefalotin; CEZ, ceftazidime; CF, ceftiofur; CHL, chloramphenicol; CIP, ciprofloxacin; COL, colistin; CPD, cefpodoxime; CRO, ceftriaxone; CT, cefotaxime; CXN, cephalexin; CZL, cefazolin; DOX, doxycycline; ERY, erythromycin; FLO, florfenicol; GT, gentamicin; KN, kanamycin; NA, nalidixic acid; NIT, nitrofurantoin; NOR, norfloxacin; OA, oxolinic acid; OXY, oxytetracycline; PEN, penicillin; PIP, piperacillin; RIF, rifampicin; S, streptomycin; SPT, spectinomycin; SXZ, sulfamethoxazole; TET, tetracycline; TI, ticarcillin; TMP, trimethoprim; T-S, trimethoprim sulfamethoxazole; VAN, vancomycin.

**Table 6:** Zoonotic bacteria associated with fish.

**Name of the pathogen**	**Disease**
*Aeromonas hydrophila*, *A. sobria*, and *A. caviae*	Skin and wound infections, systemic infections, and diarrhea
*Clostridium botulinum* and *C. perfringens*	Botulism and diarrhea
*E. coli*	Diarrhea and systemic infections
*Listeria monocytogenes*	Diarrhea and systemic infections
*Vibrio parahaemolyticus* and *V. cholerae*	Diarrhea
*Vibrio damselae*, *V. vulnificus*, *V. mimicus*, *V. fluvialis*, and *V. alginolyticus*	Skin and wound infections and systemic infections
*Salmonella*	Diarrhea and systemic infections
*Shigella*	Diarrhea and systemic infections

## Data Availability

Data sharing is not applicable to this article as no new data were created or analyzed in this study.
